# Enhanced expression of asparagine synthetase under glucose-deprived conditions promotes esophageal squamous cell carcinoma development

**DOI:** 10.7150/ijms.39557

**Published:** 2020-02-04

**Authors:** Kang Fang, Yuan Chu, Ziying Zhao, Qinfang Li, Hongqi Li, Tao Chen, Meidong Xu

**Affiliations:** Endoscopy Center, Shanghai East Hospital, Tongji University School of Medicine, Shanghai, China

**Keywords:** esophageal squamous cell carcinoma, cancer development, asparagine synthetase, glucose-deprivation

## Abstract

**Background**: Cancer cells survive and develop under nutrient deficient microenvironment caused by low blood supply. Although anaerobic metabolism could function through the enhanced uptake of glucose, other mechanisms of tolerance to glucose deficient conditions might be required.

**Materials and Methods**: Expression of asparagine synthetase (ASNS) under normal glucose and glucose-deprived conditions was examined. Cancer cell proliferation and migration were evaluated by *in vitro* and* in vivo* assays. In addition, the relationship between ASNS expression and cancer stages was also analyzed.

**Results**: Expression of ASNS was enhanced under glucose deficient conditions. *In vitro* assays indicated that ASNS could promote the proliferation and migration abilities of esophageal squamous cell carcinoma (ESCC) cells under glucose deficient condition. In mechanism, 2 critical effectors during nutrient deprivation, NRF2 and ATF4, were upregulated and demonstrated to promote ASNS expression. Clinically, high level of ASNS was significantly associated with ESCC with advanced stages and metastasis. *In vivo*, ASNS could promote tumor growth and metastasis in mouse xenograft models.

**Conclusion**: This study uncovered that glucose deprivation induces the overexpression of ASNS in ESCC cells, which in turn causes cancer cell tolerance to nutrient stress and promotes cancer development. The illustration of the mechanism sheds deep insight on how cell biology was regulated in response to the conditions of limited nutrient availability.

## Introduction

Although surgical resection improves survival of esophageal squamous cell carcinoma (ESCC), the common cancer type in Asia, many patients are not eligible for surgery due to the cancer in advanced stages^1^. The major reason for the poor outcome is due to cancer development^2^. For instance, even in the early stages, ESCC presents with lymph node metastasis, limiting the opportunities for minimal local resection, including endoscopic submucosal dissection (ESD). Thus, more effort is needed to find predictive molecular markers and illuminate the mechanism of the cancer progression.

In solid tumors, rapid growth and impaired vascular function lead to low blood supply. The combination of a lack of oxygen and a lack of nutrients causes energy deprivation^3^. Recent studies have indicated that oxygen supply <0.5% does not change cancer cell growth^3^, ^4^. It seems that hypoxia alone is insufficient to cause apoptosis. Cancer with reduced perfusion and poor oxygen supply may be subject to other stress, for instance, hypoglycemia. Glucose is an indispensable nutrient under hypoxia. When lack of energy production by aerobic metabolism, glucose uptake and glycolysis compensate is enhanced^5^, ^6^. Hypoxia causes apoptosis only in the absence of glucose^7^. These studies suggest that glucose deficiency, rather than hypoxia, might be an important factor in cancer cell death. Therefore, enhancing anaerobic metabolism is an important response to hypoxia and glucose deficiency. Under hypoxic glucose-deprived conditions, although anaerobic metabolism could work through the enhanced glucose uptake, other mechanisms to promote cancer progression might be required.

Asparagine synthetase (ASNS) is an enzyme that catalyzes the biosynthesis of L-asparagine from L-aspartate^8^. In human tissues, ASNS expression is highly regulated by the cell nutritional condition^9^. Its expression is enhanced by glucose and amino acid deprivation^10^. However, the mechanism of ASNS overexpression in the nutrient deficient condition is not clear, and it's only known that ASNS may be a key defense enzyme for cells tolerance to glucose deprivation^11^. Here, we demonstrated that high level of ASNS predicted ESCC advanced stages. Moreover, ASNS expression was enhanced by glucose deprivation *in vitro*. In mechanism, enhanced expression of NRF2/ATF4 in human ESCC cancer cells resulted in ASNS upregulation. These findings revealed a previously unidentified pathway underlying the nutritional condition dependent regulation of ASNS expression by NRF2 and ATF4 and highlighted the critical role of ASNS in ESCCs.

## Methods

### Cell lines and culture conditions

ECA109 and TE1 cells were maintained at 37 ^o^C in 5% CO2 in DMEM medium (GIBCO) supplemented with 10% FBS. In this study, different glucose concentrations of DMEM were used, from 100 mg/dl to 10 mg/dl. Two human ESCC cell lines, ECA109 and TE1 were purchased from the Institute of Biochemistry and Cell Biology of the CAS (Shanghai, China). DMEM containing 100 mg/dL glucose was used as the normal glucose condition and glucose-free DMEM was used as the glucose-deprived condition. Then, DMEM with different glucose concentrations was prepared from glucose-free DMEM and sterile glucose powder.

### Cell proliferation and transwell assays

For evaluating the proliferation of ESCC cells, MTT assays were done. Briefly, a total of 1 × 10^3^ cells were seeded in 96-well plates. Then, 10 μL MTT reagent and 90 μL DMEM were added and the cells were incubated. After 2 h of incubation, the absorbance of cells at 450 nm was measured using a microplate reader. For evaluating the migration of ESCC cells, a 24-well transwell plate was used to measure each cell line's migratory ability. 5×10^4^ cells were plated in the top chamber lined with a non-coated membrane and this assay was performed according to the standard procedure. Cells on the lower surface of the insert were Giemsa stained and images from three representative fields of each membrane were taken using a light microscope (100x). The number of migratory or invasive cells was counted. Each experiment was repeated five times.

### RNA extraction and real-time PCR assay

Total RNA was isolated using TRIzol reagent according to the manufacturer's instructions. TaqMan real-time PCR assays for ASNS and ATF4 were from Takara Bio. The primers were summarized in Supplementary table 1. All reactions, including the no template controls, were run in triplicate. After the reactions were completed, the CT values were determined using fixed threshold settings.

### Immunoblotting analysis

Proteins were extracted from cultured cells, followed by immunoblotting with the primary antibodies. The protein concentration was determined through Bradford assay. Proteins were separated by SDS-PAGE, transferred onto PVDF membrane (Millipore Corporation) and probed with the indicated antibodies. Antibodies that recognize ASNS (ab40850), NRF2 (ab62352), ATF4 (ab184909), and Actin (ab8227) were purchased from Abcam.

### Chromatin immunoprecipitation assay

A Chromatin Immunoprecipitation assay was performed using an Upstate Biotechnology kit. Briefly, cells were cross-linked with 1% formaldehyde plus 1.5 mmol/L ethylene glycol bis (succinimidyl succinate) at room temperature. Cross-linked chromatin was sonicated and precipitated with antibodies against transcription factor. Quantitative real-time PCR was used to measure the amount of bound DNA, and the value of enrichment was calculated according to the relative amount of input and the ratio to IgG. The primers covering NRF2 binding site of ATF4 gene promoter region and the primer covering ATF4 binding site of ASNS gene promoter region were summarized in Supplementary Table 1.

### Immunohistochemistry

This study was approved by the institutional review board of East Hospital, Tongji University. Immunohistochemical analysis was conducted as described previously 12. Consecutive sections of formalin-fixed, paraffin-embedded (FFPE) tumors that were obtained from ESCC patients were subjected to immunohistochemical analysis. Briefly, 4 μm thick sections were deparaffinized in xylene and gradually rehydrated in alcohol solutions. After normal antigen retrieval, IHC staining was performed following the manufacturer's instructions. Subsequently, anti-ASNS rabbit monoclonal antibody (ab40850) was used at 4°C. Slides were counterstained with light hematoxylin, dehydrated, and cover-slipped.

### Animals

ECA109 cells, which were infected with anti-ASNS-LV or anti-NC-LV, were harvested, suspended and injected into the tail vein of each male nude (nu/nu) mouse (2×10^6^ viable tumor cells/mouse). In addition, cells subcutaneously injected into the right flanks of each mouse (5×10^6^ viable tumor cells/mouse). The animals were randomly divided into 2 groups (control and treated groups, 8 mice per group). At 6 weeks, the lungs of nude mice and subcutaneous tumors were removed. Tumors and lungs were fixated in 10% phosphate-buffered formaldehyde, and embedded in paraffin for further investigation. All animal experiments were performed and approved by the animal care and use committee of Tongji University.

### Statistical analysis

All experiments were performed in triplicate. Differences between groups were calculated using Student's t test, Chi-square test, or Fisher's exact test. The SPSS software program (version 19.0; SPSS Inc.) was used for statistical analyses.

## Results

### Expression of ASNS under normal glucose/glucose-deprived conditions

**Figure 1:**
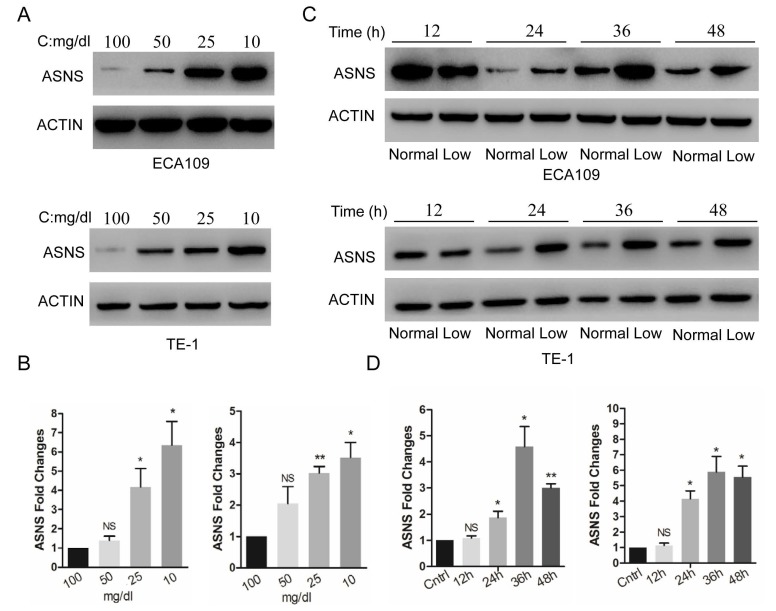
Expression of ASNS under different conditions. A, ECA109 and TE1 cells were incubated under different glucose conditions from 100 mg/dl to 10 mg/dl. Immunoblotting assay was employed to detect the protein level of ASNS. B, Real-time PCR was employed to detect the mRNA level of ASNS under different glucose conditions. C, ECA109 and TE1 cells were incubated under normal glucose conditions (100 mg/dL, normal) and glucose-deprived conditions (10 mg/dL, low). After incubation for different time course, the protein level of ASNS was analyzed by immunoblotting assay. D, After incubation under normal glucose conditions (normal) and glucose-deprived conditions (low) for different time course, the mRNA level of ASNS was analyzed by real-time PCR. *, P < 0.05.

The expression of ASNS protein and mRNA were examined by immunoblotting and real-time PCR in 2 ESCC cell lines, ECA109 and TE1. These cells were cultured in different glucose concentrations of DMEM, from 100 to 10 mg/dl. Overall, the low concentrations of medium significantly increased the ASNS protein levels (Fig. 1 A), as well as mRNA levels (Fig. 1 B), especially under the concentration of 10 mg/dl. Furthermore, in both cells, ASNS protein was increased after 12 h incubation under glucose-deprived conditions (10 mg/dl, Fig. 1 C). In addition, the high level of ASNS mRNA was sustained in time course experiments (from 12 h to 48 h; Fig. 1 D).

### Glucose-deprived condition induces NRF2/ATF4 expression and promotes ASNS upregulation

**Figure 2:**
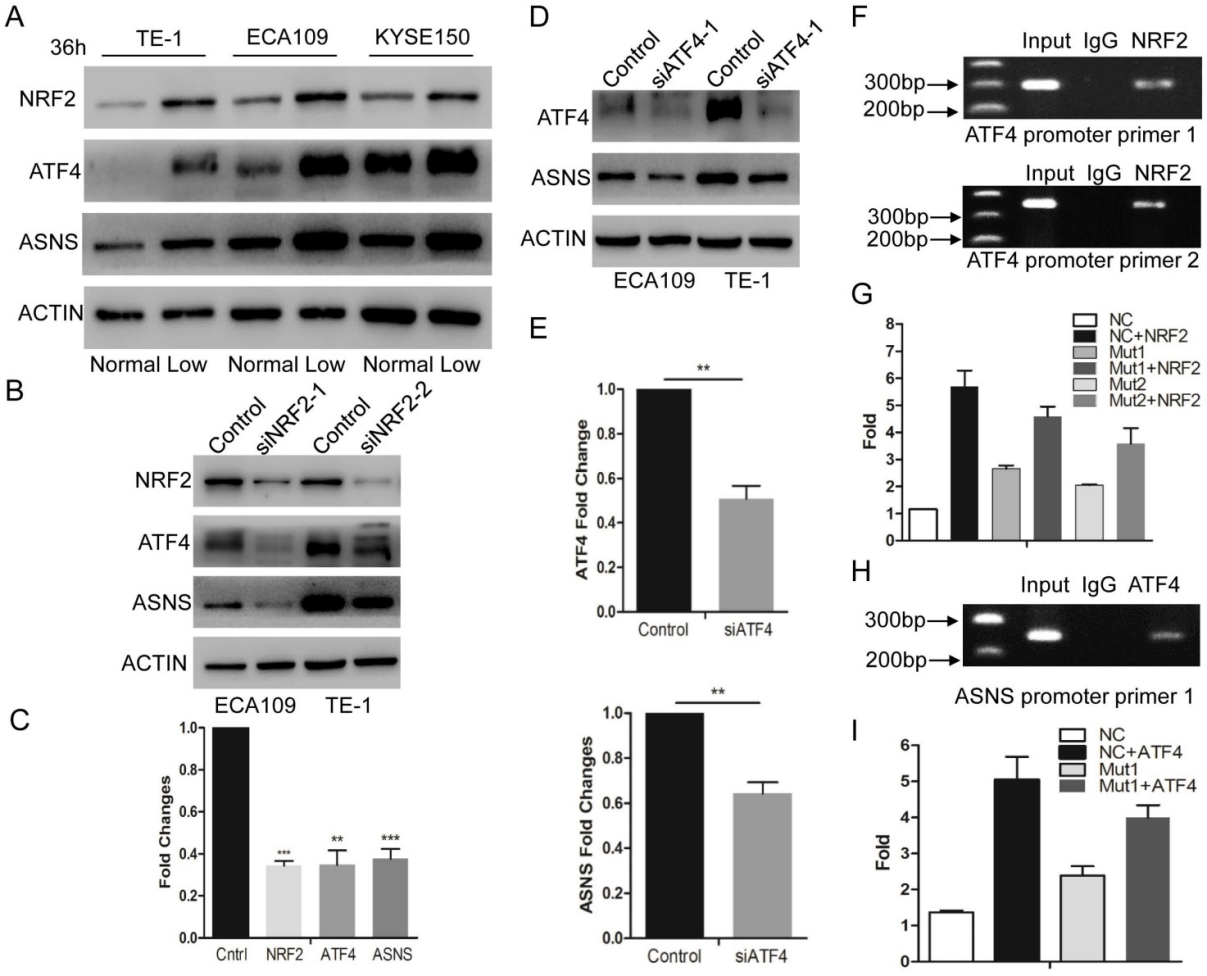
Glucose-deprived condition induces NRF2/ATF4 expression and promote ASNS upregulation. A, ECA109, TE1 and KYSE150 cells were incubated under normal glucose conditions (100 mg/dL, normal) and glucose-deprived conditions (10 mg/dL, low). After incubation for 36 h, the protein level of NRF2, ATF4 and ASNS was analyzed by immunoblotting assay. B, Immunoblotting analysis showed that NRF2 affected the protein levels of ATF4 and ASNS. ACTIN was used as a protein loading control. C, Real-time PCR showed that NRF2 affected the mRNA levels of ATF4 and ASNS. D, Immunoblotting analysis showed that ATF4 affected the protein levels of ASNS. E, Real-time PCR showed that ATF4 affected the mRNA levels of ASNS. F, Analysis of the physical association of regions of the ATF4 promoter with NRF2 by ChIP assays. G, Luciferase reporter gene assays showed the transactivation activity of the indicated serial ATF4 promoter fragments in the indicated ECA109 cells. H, Analysis of the physical association of regions of the ASNS promoter with ATF4 by ChIP assays. I, Luciferase reporter gene assays showed the transactivation activity of the indicated ASNS promoter in the indicated ECA109 cells. * represents P<0.05, ** represents P<0.01.

According to recent studies, NRF2 and ATF4 are critical effectors during nutrient deprivation 1, 13, 14. NRF2 is a transcription factor response to cellular stress signals. It also regulates several transcriptional factors including ATF4^15^. In the present study, we tested whether glucose deprivation would stimulate the expression levels of NRF2 and ATF4. We examined their expression levels by immunoblotting in 3 ESCC cell lines and confirmed that these 2 protein levels were upregulated under glucose-deprived condition (10 mg/dl, Fig. 2 A). Furthermore, we used siRNAs to interfere NRF2 expression and found that down-regulated NRF2 decreased the expression of ATF4 and ASNS under glucose-deprived conditions (Fig. 2 B and C). Simultaneously, siRNAs for ATF4 suppressed the ASNS overexpression in both ECA109 and TE1 cell lines (Fig. 2 D and E). To further study the direct mechanism under this process, ChIP assay was done to verify the binding site of NRF2 in the ATF4 (Fig. 2 F). Additionally, luciferase assays demonstrated that NRF2 could increase the ATF4 transcriptional activation (Figure 2G). Moreover, ChIP assay verified the binding site of ATF4 in ASNS promoter regions (Fig. 2 H) and ATF4 could increase the ASNS transcriptional activation (Figure 2I). These data indicated that the ASNS expression under glucose-deprived conditions is induced by the NRF2/ATF4 axis.

### Silencing of ASNS inhibits proliferation and migration of cancer cells

**Figure 3:**
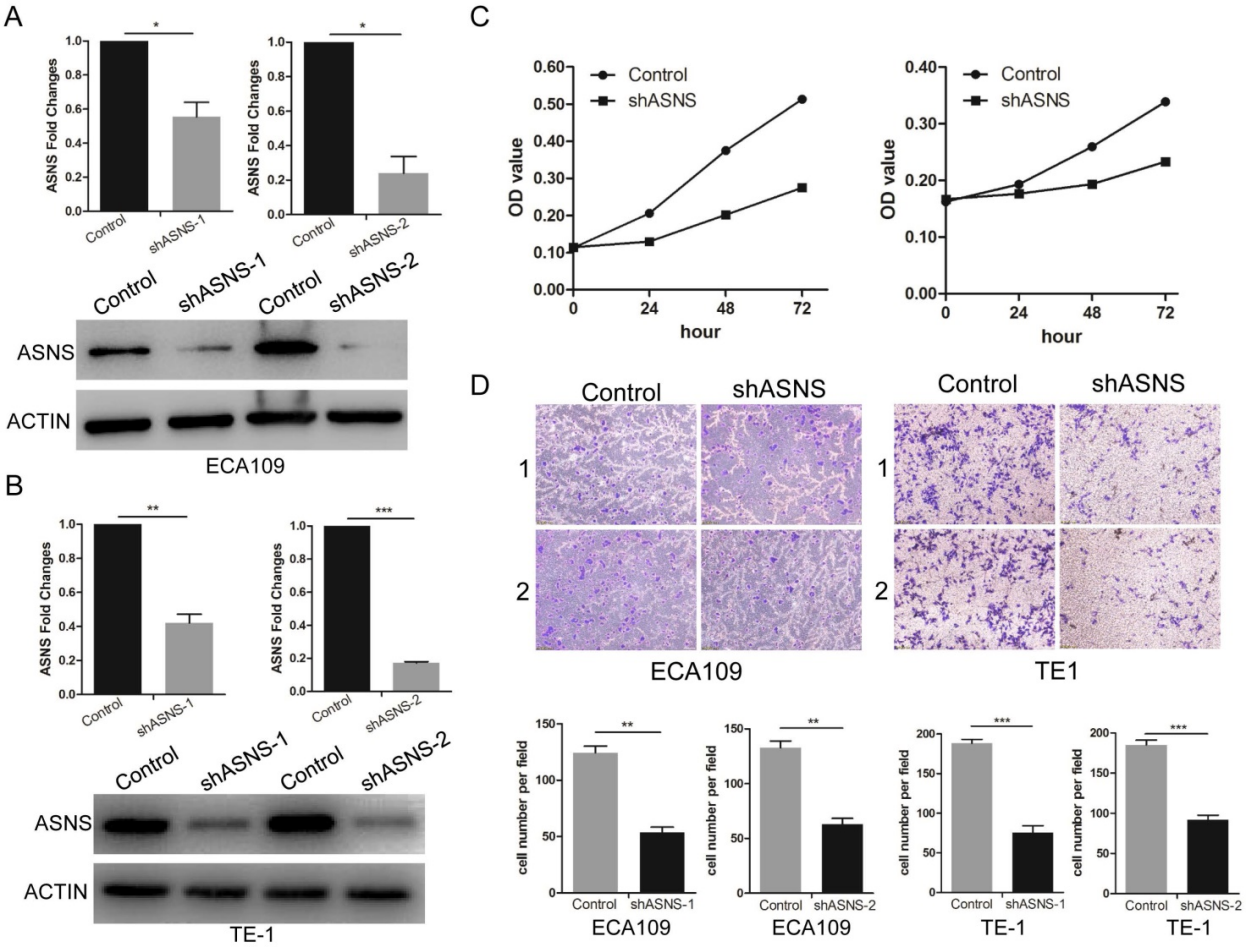
The effect of ASNS on ESCC cell migration and proliferation. A, The effect of ASNS-shRNAs was examined in ECA109 cells by real-time PCR and immunoblotting assay. B, The effect of ASNS-shRNAs was examined in TE-1 cells by real-time PCR and immunoblotting assay. C, ECA109 and TE-1 cells were transfected with ASNS-shRNAs. The proliferation ability of ECA109 and TE-1 cells was examined under glucose-deprived condition. D, ECA109 and TE-1 cells were transfected with ASNS-shRNAs. The migration ability of ECA109 and TE-1 cells was examined under glucose-deprived condition. * represents P<0.05, ** represents P<0.01, and *** represents P<0.001; student's *t* test.

To further study the ASNS effect on the functions of ESCC cells, transwell and MTT assays were performed. ShRNAs were employed to knockdown ASNS expression in ECA109 and TE1 cells. Subsequently, both protein and mRNA levels of ASNS were significantly down-regulated by shRNA-ASNS (Fig. 3 A and B). The results of MTT assays indicated that knockdown of ASNS decreased the cell proliferation in ECA109 and TE1 cell lines (Fig. 3 C). Furthermore, transwell assays indicated that knockdown of ASNS decreased the ECA109 and TE1 cell migration under glucose-deprived conditions (Fig. 3 D).

### ASNS upregulation is associated with ESCC development

**Figure 4:**
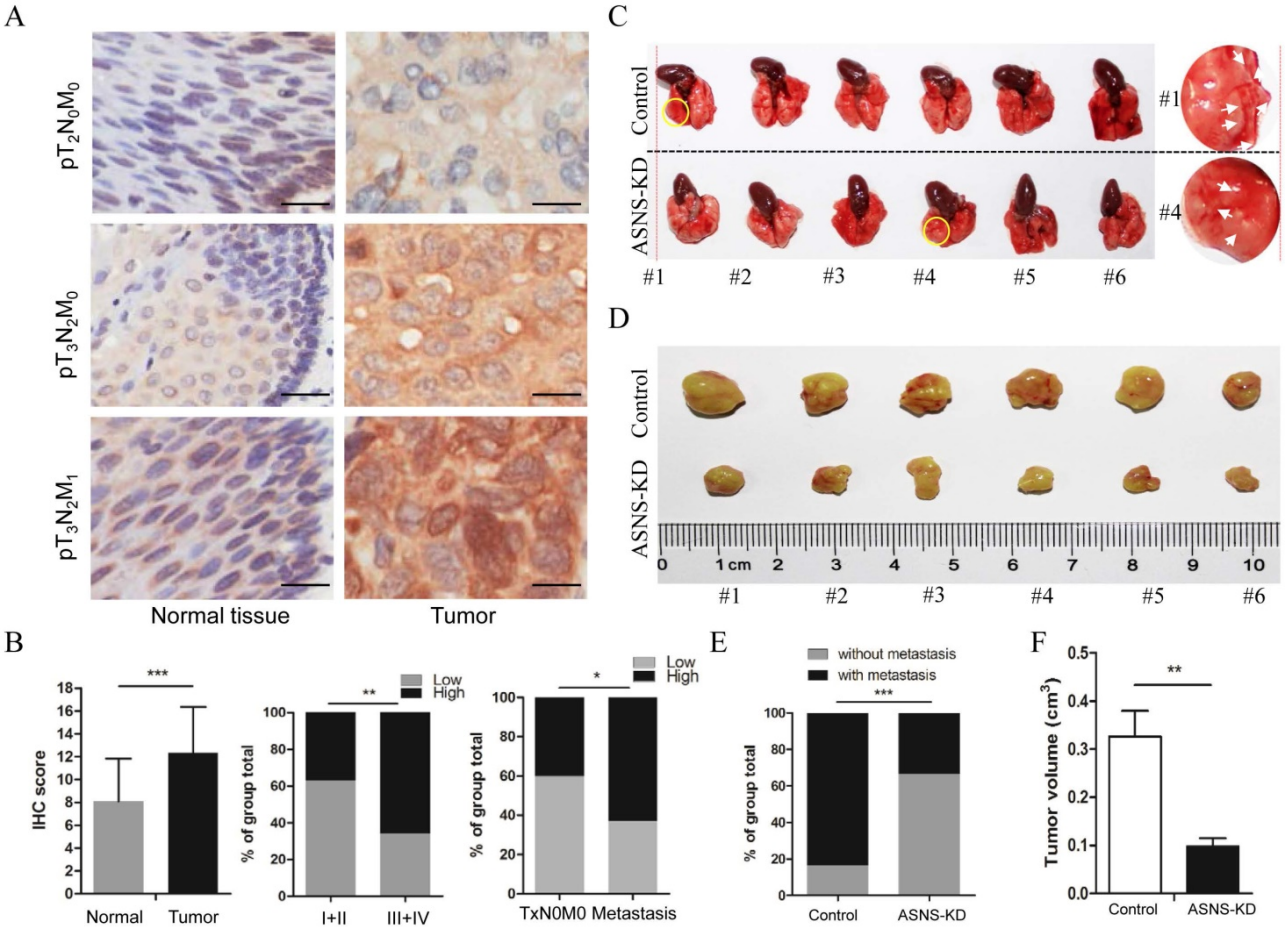
ASNS expression was associated with ESCC development. A, ASNS expression level in ESCC specimens was significantly higher than that in normal tissue specimens. B, ASNS expression level was significantly higher in ESCC with advanced stages specimens. C, The lungs of nude mice as tumor metastasis model. D, The tumors of nude mice as subcutaneous tumor model. E, The number of visible metastatic lesions in the lung was measured. F, The subcutaneous tumor size was measured. * represents P<0.05, ** represents P<0.01, and *** represents P<0.001; student's *t* test.

To evaluate the clinical meaning of ASNS in ESCC patients, ASNS protein expression was assessed in 85 primary ESCC tissues by IHC. We found that the ASNS protein level was significantly higher in ESCC tissues than that in normal tissues (Fig. 4 A). Then, we analyzed the correlation of clinicopathologic parameters with ASNS level in ESCC. We found that ASNS protein level was higher in Stage III+IV cancers than that in Stage I+II cancers (Fig. 4 B). Meanwhile, ASNS expression was higher in ESCC with metastasis than that without metastasis (Fig. 4 B).

Furthermore, we studied the ASNS effect on cancer growth and metastasis by mice modes. For primary cancer model, nude mice were injected subcutaneously with ASNS knockdown or negative control cells. For cancer metastasis model, nude mice were injected intravenously in the tail vein. As a result, down-regulated ASNS significantly decreased the subcutaneous tumor size or number of lung metastasis nodes (Figure 4 C-F).

## Discussion

ESCC typically presents with metastasis even in the early stages, limiting the opportunity for curative local surgical resection and prevention of cancer progression. Therefore, it is important to understand the genetic factors of the cancer development and the risk of metastasis. This study provided evidence on the role of ASNS in modulating the developmental potential of ESCC. With clinical samples, the ASNS expression was higher in the ESCC tissues and moreover, the protein level was associated with advanced stages and metastasis. Both *in vitro* and *in vivo* studies also supported the above hypothesis and indicated that ASNS functioned as a promoter for ESCC cell growth and migration.

Cancer cells face a challenge in rapid proliferation under limited nutrient conditions. The mechanisms under how oncogenes help cancer cells tolerance to nutrient stress are not fully clear. In this study, we found that glucose deprivation increases the ASNS expression in ESCC cells, which confirmed the findings in previous reports 5, 11, 16. Furthermore, ASNS was demonstrated to regulate ESCC cell proliferation and migration. ASNS promotes the proliferation and migration abilities of ESCC cells under glucose deficient condition. It is reported that intracellular asparagine expression is lower than other amino acids 17, however, this level is enough to support cancer cell proliferation, which allows asparagine to be a sensitive gauge of nutrient availability. Thus, ASNS overexpression can promote tumor growth under nutrient stress. ASNS is identified as a molecular predictor in ESCC advanced stages and metastasis. Moreover, ASNS knockdown could be used as an ESCC therapeutic strategy, which is established in other cancers^18^. This represents a potential treatment approach for ESCC patients.

In this study, NRF2-ATF4 axis was identified as the key transcription factors regulated by nutrient condition to promote ASNS expression and support ESCC development. During nutrient stress, NRF2 overexpression could support amino acid homeostasis through ATF4 19. NRF2 has been demonstrated to modulate cell metabolism. The NRF2-ATF4 axis is important for the asparagine biosynthesis. Our results demonstrate that NRF2 was upregulated under glucose deprivation and increased the expression levels of its downstream target genes under a transcriptional mechanism. In addition, this pathway was required for the activation of ATF4, another key transcription factor. NRF2 and ATF42 are upregulated under nutrient deprivation and promote ASNS expression. Finally, ATF4 mediated ASNS overexpression by NRF2 upregulated in nutrient stress.

## Conclusion

Glucose deprivation induces the overexpression of ASNS in ESCC cells. ASNS overexpression causes cancer cell tolerance to nutrient stress and cancer development. The role of ASNS is identified as a molecular predictor in ESCC advanced stages and metastasis. The illustration of the mechanism by which ASNS expression is increased under nutrient-limited conditions sheds insight on how cell biology is regulated in response to microenvironmental changes; the results of this study provide a molecular aspect for developing therapeutic benefits of targeting NRF2/ATF4/ASNS axis in ESCC.
