# Supplementary material for: Enhanced expression of asparagine synthetase under glucose-deprived conditions promotes esophageal squamous cell carcinoma development
Source: Int J Med Sci. 2020 Feb 4;17(4):510–6. doi: 10.7150/ijms.39557 (PMC7053306; doi:10.7150/ijms.39557)
Supplement: Supplementary file 1 — Supplementary table. [file ijmsv17p0510s1.pdf]

Supplementary Table 1 Primers for real-time PCR assays

ASNS	forward	5'-CTGCACGCCCTCTATGACA-3'
	reverse	5'-TAAAAGGCAGCCAATCCTTCT-3'
ATF4	forward	5'-ATGACCGAAATGAGCTTCCTG-3'
	reverse	5’-GCTGGAGAACCCATGAGGT-3'
ASNS (for ChIP)	forward	5’- CAGGGTGATGTGGCGGGCTGAGG -3’
	reverse	5’- TTAAACAGGCGCACTGAGACGCA -3’
ATF4 (for ChIP) 1	forward	5’-GCGGGCGGGAGGAGACGGTCACGTG-3’
	reverse	5’-ATACGCCATGGTGGCCGTGGACCCT-3’
2	forward	5’-GGGGGCAGGAGCCAGCGCGATCGTG-3’
	reverse	5’-GAGTTTTTATTGTGAAGGCCGAGGA-3’
